# Poly (3,4-Ethylenedioxythiophene) (PEDOT) Nanofibers Decorated Graphene Oxide (GO) as High-Capacity, Long Cycle Anodes for Sodium Ion Batteries

**DOI:** 10.3390/ma11102032

**Published:** 2018-10-19

**Authors:** Zejun Pu, Penglun Zheng, Yu Zhang

**Affiliations:** 1 College of Materials Science and Engineering, Sichuan University of Science & Engineering, Zigong 643000, China; puzejunuestc@163.com; 2 High Temperature Resistant Polymer and Composites Key Laboratory of Sichuan Province, University of Electronic Science and Technology of China, Chengdu 610054, China; 3 School of Materials Science and Engineering, Nanyang Technological University, 50 Nanyang Avenue, Singapore 639798, Singapore

**Keywords:** graphene oxide, in-situ polymerization, conductive polymer, hybrid, sodium ion battery

## Abstract

Conductive Poly (3,4-ethylenedioxythiophene) (PEDOT) nanofibers are uniformly deposited on ultrathin graphene oxide (GO) nanosheets via a simple and effective in situ polymerization process under ambient conditions. The as-prepared samples are characterized by field-emission scanning electron microscopy (FE-SEM), transmission electron microscopy (TEM), Raman spectra, Fourier transforms infrared spectra (FTIR), and electrochemical measurements. The results indicate that the as-obtained PEDOT–GO hybrid (GDOT) achieves excellent sodium storage properties. When explored as a new inorganic/polymeric electrode for sodium ion batteries (SIBs), the GDOT exhibits a high reversible capacity (338 mAh g^−1^), good cycling stability (234 mAh g^−1^ after 400 cycles), and excellent rate capabilities (e.g., 62 mAh g^−1^ at 30 A g^−1^) due to their ultrathin structure as well as conductive network. This easily scale-up-able and effective strategy shows great potential for large-scale energy applications.

## 1. Introduction

Lithium ion batteries (LIBs) have been the leading energy storage technology for portable devices and electrical vehicles (EVs) during the past two decades. However, the challenges for its large-scale application are the scarcity of lithium source as well as its high cost [1,2,3]. Sodium ion batteries (SIBs) have attracted emerging scientific attention as an alternative to LIBs as next-generation energy storage technology due to the abundance (~2.5% in the Earth’s cost), low cost, as well as similar chemistry properties of sodium to lithium [4]. However, SIBs are confronted with the challenges in terms of low energy density, low power density, and inferior cycle life. To address these issues, intensive work has been performed on developing electrode materials, especially cathode materials [5]. However, research on the anode materials for SIBs is still in its infancy due to the unsuccessful adaptation of LIBs electrodes to SIBs [6]. For example, graphite as the most common anode materials for commercial LIBs has been demonstrated electrochemically inactive with a very low capacity [7,8]. In addition, other well-developed anodes for LIBs, such as alloying-type materials (Sn [9], Sb [10], P [11], etc.) and transition metal oxides/sulphides (CoO [12], FeS_2_ [13], etc.) are hindered by the large volume change and sluggish sodiation kinetics, leading to inferior rate capability as well as poor cycling stability. Therefore, developing appropriate anode materials for SIBs with high rate capability and excellent cycling life still requires further effort before putting into practical application.

Carbonaceous materials (e.g., carbon nanotube [14], expanded graphite [15], and reduced graphene oxide (rGO) [16], etc.) are considered as potential anodes for SIBs owing to its naturally abundant distribution and environmental benignity. Moreover, they are free from huge volume change compared with those conversion and alloy type anodes during the Na ion (de-) insertion [17]. However, challenges remain regarding their unsatisfactory electrochemical properties as well as large-scale applications. For example, hard carbon exhibits poor rate capability due to its lower electronic conductivity and limited nanopore-filling sodium ion process compared to other carbon materials like rGO [18]. Expanding graphite has been reported as an effective strategy towards the inactive pristine graphite for SIBs, but its capacity falls from 284 mAh g^−1^ at 20 mA g^−1^ to 91 mAh g^−1^ at 200 mA g^−1^. In addition to hard carbon and graphite, rGO with a novel 2D structure has attracted much attention as a promising Na-ion storage material due to its outstanding electronic conductivity, high surface-to-volume ratio, and good flexibility [16]. Nonetheless, rGO sheets tend to restack, which leads to difficult slurry processing as well as poor capacity retention during cycling [19]. In addition, rGO are generally prepared by thermal or chemical reduction process of GO, which needs multiple operation process, high-temperature conditions, or specific atmosphere [20]. Alternatively, graphene oxide (GO) can be prepared in a large scale by the well-developed Hummers’ method [21]. It is known that low cost and scalability of electrode synthesis are crucial for practical applications. Furthermore, GO readily dissolves in N-Methyl-2-pyrrolidone (NMP) or aqueous solvents due to its hydrophilic property, which are favorable features for electrode processing [22]. However, GO is electrically insulating, and few studies have been reported on the development of GO as the anode for SIBs. Therefore, we are inspired to develop approaches to prepare efficient GO-based anode for SIBs. 

Nanoscale surface engineering of electrode materials plays a key role in the interfacial reactions between electrodes and electrolyte. Herein, we report effective surface engineering of GO by homogeneously bottom-up growing conductive polymer-PEDOT (Poly (3,4-ethylenedioxythiophene)) nanofibers on GO nanosheets via a facile in situ polymerization process and study its sodium storage properties. The resulting PEDOT–GO hybrid (GDOT) architecture possesses the following merits: (1) ultrathin configuration endowing short ion diffusion path; (2) much improved electrical conductivity facilitating fast charge transfer; (3) high specific surface areas providing sufficient active sites; and most importantly (4) easily scale-up-able methodology under ambient conditions. Benefiting from these unique characteristics, the as-synthesized GDOT electrodes present a high reversible capacity of 338 mAh g^−1^ at 100 mA g^−1^, preferable rate (e.g., 136 mAh g^−1^ at 10 A g^−1^) and cycling performance (234 mAh g^−1^ after 400 cycles at 100 mA g^−1^) when working as the anode for SIBs.

## 2. Experimental Section

Synthesis of GO: high-quality graphite oxide was prepared from natural graphite powder (325 mesh) according to the modified Hummers’ method as reported by Du [23]. Firstly, 6 g graphite powder was dispersed in 48 mL 98% H_2_SO_4_ followed by the addition of 10 g K_2_S_2_O_8_, and 10 g of P_2_O_5_ to form a homogeneous solution with a physical dispersion technology, which utilized a ball milling machine by high-speed circulation (15,000 rpm, 10 min). Then, the mixture solution was transferred to a 100 mL three-necked round bottom flask and maintained at 80 °C for 4.5 h. After cooling naturally to room temperature, the preoxidized products were obtained by centrifuging and rinsed for 3–4 times to remove residuum. In addition, the preoxidized products were dried under vacuum at 60 °C for 12 h. After that, the as-prepared preoxidized products were homogeneously dispersed in 240 mL 98% H_2_SO_4_ solution by ultrasonic dispersion technology, 30 g of KMnO_4_ was then slowly added into the above solution with a low temperature (<20 °C) to avoid overheating and explosion. Subsequently, the temperature of solution was increased to 35 °C and kept for another 2 h. After 2 h, 1400 mL H_2_O was slowly added to dilute the above solution, and 800 mL 30% H_2_O_2_ was trickled into the mixture solution to completely react with the superfluous KMnO_4_. After the reaction, the bright yellow mixture solution was filtered using a 0.2-μm PTFE filter membrane and washed by distilled (DI) water for 3 times. Ultimately, the neutral GO aqueous solution was freeze-dried to remove the remaining water inside and the high-quality GO powder was obtained.

Fabrication of GDOT: the as-prepared high-quality GO (20 mg) was further homogeneously dispersed in a mixed solution of DI water (10 mL) and ethanol (30 mL) via a short sonication, following by adding 40 µL of EDOT with the help of magnetic stirring for 15 min, and simultaneously 270 mg FeCl_3_ was dissolved in 10 mL ethanol. The FeCl_3_/ethanol solution was added slowly into the above solution, and then the mixed solution was first maintained violently stirring (2000 rpm) for 5 min and then maintained slow stirring (400 rpm) for another 20 min and 40 min, respectively. Ultimately, the PEDOT–GO hybrids, namely GDOT, were obtained after centrifuging and washing with DI water and ethanol, respectively.

Measurement and Characterization: Field-emission scanning electron microscopy (FE-SEM, JEOL JSM-7600F, Tokyo, Japan) and Transmission electron microscopy (TEM, JEOL-2100F, Tokyo, Japan) were applied to observe the micro-morphology of the as-prepared GO, GDOT, and pure PEDOT fibers, respectively. Raman spectra of GO was recorded from 600 to 2500 cm^−1^ on a micro-Raman spectrometer (Witec CRM200, WITec GmbH, Germany). Fourier transform infrared (FTIR, NICOLET 6700, Waltham, Massachusetts, America) was utilized to investigate the structure of pure GO and GDOT between 550 cm^−1^and 3200 cm^−1^. The preparation of electrode slurries and assembly of coin cells (2032) were carried out as described in the previous literature [6]. For a direct comparison, the electrochemical activities of the Single-Walled Carbon Nanotubes (SWCNT) were investigated and are presented in Figure S1 (Supporting Information). The charge and discharge tests were carried out by a LAND battery tester with a voltage window of 0.01–3.0 V. Cyclic voltammetry (CV) curves and electrochemical impedance spectroscopy (EIS) measurements for GDOT and GO electrodes were carried out in the AC frequency (from 1 MHz to 0.01 Hz) using a Bio-Logic SP-150 model potentiostat.

## 3. Results and Discussion

**Figure 1:**
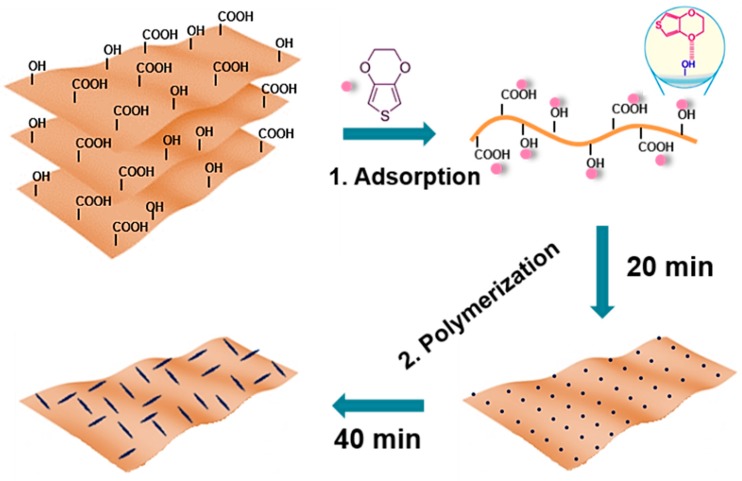
Schematic diagram of the fabrication of process for GDOT architectures: (1) Adsorption of EDOT on the surface of GO by hydrogen bonding. (2) In situ polymerization of PEDOT nanofibers on GO with controlled reaction time under ambient conditions. PEDOT, Poly (3,4-ethylenedioxythiophene); GO, graphene oxide; GDOT, PEDOT–GO hybrid.

GO nanosheets were obtained by the well-developed Hummers’ method. The preparation of GDOT nanosheets involves the following steps as shown in Figure 1. In brief, GO was dispersed into the mixture of DI water and ethanol via sonication. Subsequently, 3,4-ethylenedioxythiophene (EDOT) molecules were added and absorbed onto the surface of GO by hydrogen bonding between the functional groups of GO surface and the oxygen atoms in EDOT monomers. Ultimately, the PEDOT fibers were uniformly decorated onto GO via in situ polymerization with controlled reaction time under ambient conditions. 

**Figure 2:**
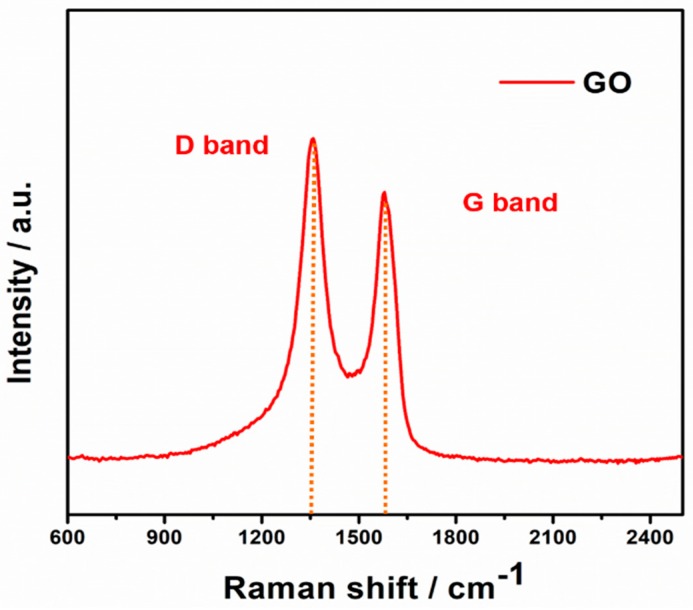
Raman spectra of GO with two prominent peaks at 1365 cm^−1^ and 1597 cm^−1^, respectively.

**Figure 3:**
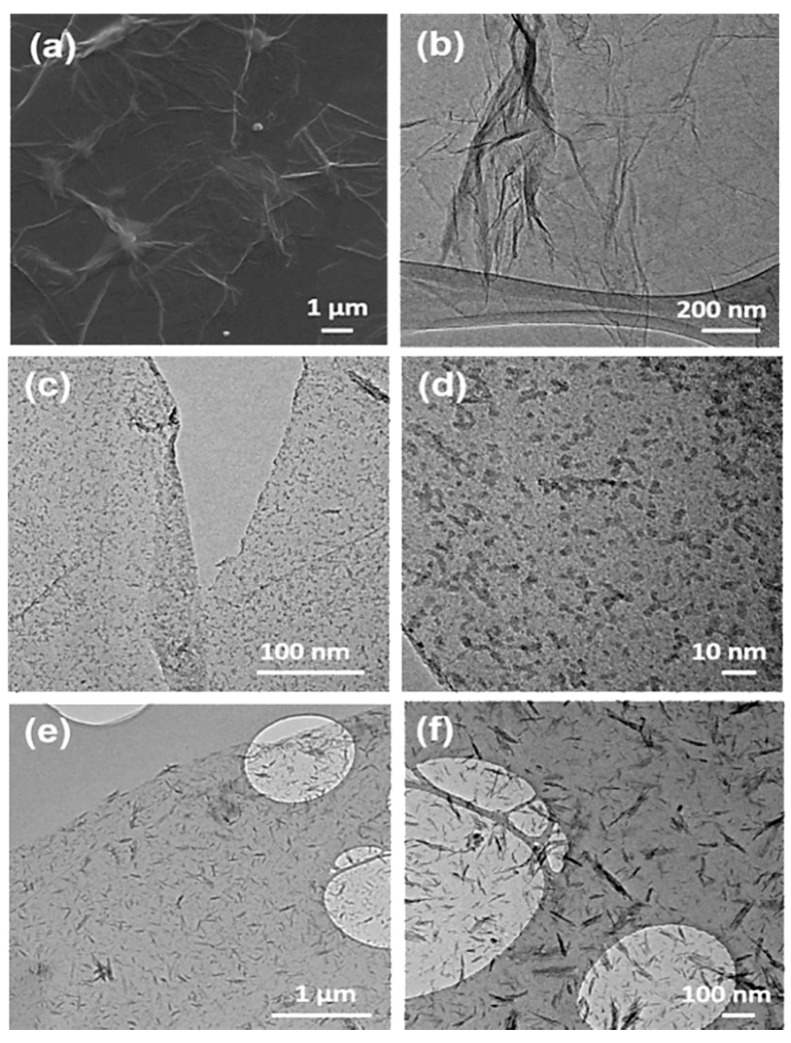
Characterizations of GO and GDOT: FE-SEM (**a**) and TEM (**b**) images of GO; TEM images of GDOT obtained within reaction time of 20 min (**c**,**d**) and 40 min (**e**,**f**).

The as-obtained GO was characterized by Raman spectra, FE-SEM, and TEM, respectively. Figure 2 displays the Raman spectra of pure GO, two prominent broad peaks at around 1365 cm^−1^ and 1597 cm^−1^ can be obviously observed, which can be ascribed to the disorder induced D band and the first-order scattering of E_2g_ allowed G band [24]. Figure 3a,b shows the FE-SEM and TEM images of GO, respectively. As shown in Figure 3a, the GO on silicon wafer shows smooth and planar surface with several micrometer in lateral size. The TEM image (Figure 3b) shows a typical transparent nanosheet, proving the ultrathin nature of GO. Then, the GDOT hybrid nanosheets were synthesized by in situ polymerization of EDOT monomers absorbing on GO in the presence of FeCl_3_. The structural evolution was examined by controlling the reaction time. It is observed that uniform PEDOT nanodots are firstly grown on the GO surface within 20 min (Figure 3c). The size of PEDOT nanodots could be determined as ca. 2 nm by the high-magnification TEM image (Figure 3d). PEDOT fibers (length: ≈100 nm; diameter: ≈10 nm) are successfully formed within 40 min, as shown in Figure 3e,f. 

**Figure 4:**
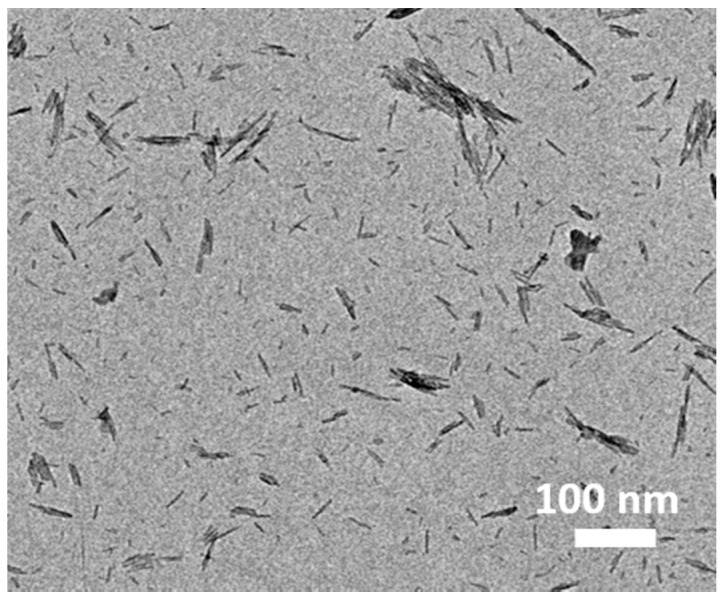
TEM image of the pure PEDOT fibers.

Furthermore, the PEDOT fibers were prepared without adding GO, and their TEM image is presented in Figure 4. The results suggest from another point of view that the PEDOT fibers were uniformly deposited on ultrathin graphene oxide (GO) nanosheets by a facile in situ polymerization process.

**Figure 5:**
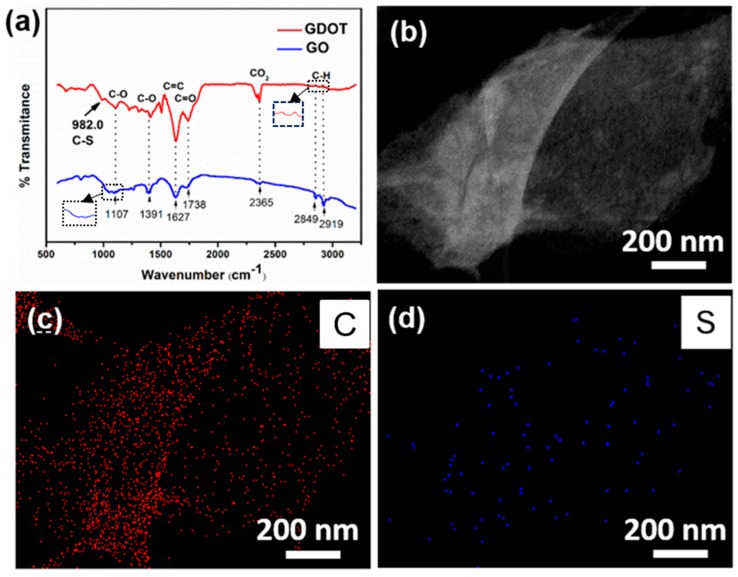
(**a**) FTIR measurements of GDOT and GO; (**b**) HAADF-STEM image of GDOT; and (**c**,**d**) the corresponding element mapping.

To further confirm the presence of PEDOT, FTIR measurement was performed on the GO and GDOT hybrid. In Figure 5a, the same peaks from both samples located at 1107, 1391, 1627, 1738, 2365, 2849, and 2919 cm^−1^ belonging to the C–O, C–O, C=C, C=O, CO_2_ and C–H bond vibrations, respectively, which could originate from graphene oxide or PEDOT [25]. It’s worth noting that a bond at 982.0 cm^−1^ for the GDOT sample can be observed, which belongs to the C-S bond vibration of PEDOT, while it is missing in GO. This indicates the successful growth of PEDOT on GO nanosheet. Furthermore, the high-angle annular dark-field scanning TEM (HAADF-STEM, Figure 5b) image and their corresponding element mapping (Figure 5c,d) show the uniform distribution of C and S element in a typical nanosheet, further confirming the presence of PEDOT in GDOT hybrid.

**Figure 6:**
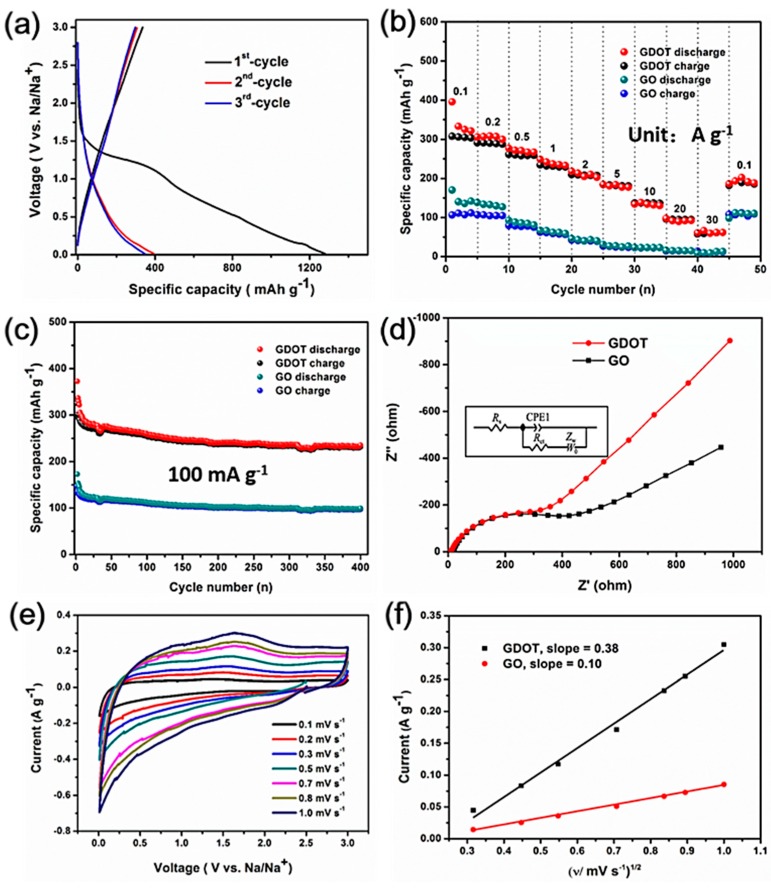
Electrochemical activity of GDOT and GO electrodes for SIBs: (**a**) The first three cycles discharge–charge curves of the GDOT electrode; (**b**) rate capability at different current densities from 0.1 to 30 A g^−1^; (**c**) cycling performance at 0.1 A g^−1^; (**d**) the electrochemical impedance spectra measurements (inset: fitted equivalent circuit); (**e**) CV curves of GDOT electrode at different scan rates from 0.1 to 1.0 mV s^−1^; and (**f**) linear relationship of the cathodic peak current (i_p_) and the square root of scan rate (ν^1/2^).

**Figure 7:**
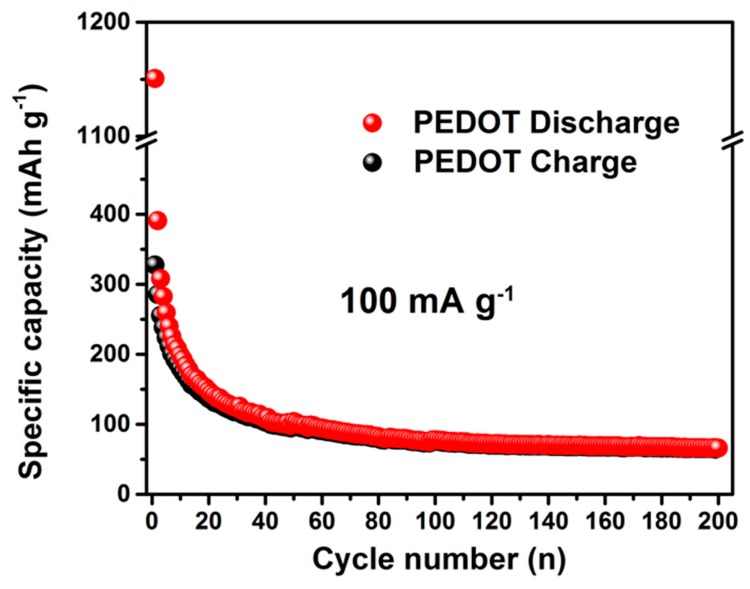
Cycling performance of the pure PEDOT electrode for SIBs at a current density of 100 mA g^−1^.

The sodium storage properties of the GDOT hybrid were investigated based on the standard half-cell configuration with sodium foil as the counter/reference electrode. Figure 6a shows the initial three cycles of the discharge-charge curves for the GDOT electrode at a current density of 100 mA g^−1^ within the potential window of 0.05 to 3.0 V versus Na/Na^+^. During the initial discharging process, a plateau at approximately 1.25 V was observed and disappeared in the following cycles, which was related to the formation of solid electrolyte interphase (SEI) on GDOT surface and the reaction between the functional groups (e.g., –OH, –COOH, etc.) on GO with Na^+^. For the charging process, no apparent plateau was recorded, indicating that Na^+^ ion extraction from the electrode has no specific voltage range. Slightly higher working potentials than hard carbon and no obvious voltage hysteresis are observed in the discharge and charge profiles, suggesting the possible alloying reaction during sodiation and desodiation process [26]. Initial discharge and charge capacities of 1278 mAh g^−1^ and 338 mAh g^−1^ were achieved, respectively. The resulting relatively low coulombic efficiency (CE) could be due to the SEI formation and other irreversible reactions between the active groups on GO with Na^+^, which is similar to those carbon-based materials reported previously [8]. During the following two cycles, the discharge–charge curves are almost overlapping, implying its good reversibility. The rate capability is a crucial aspect for high-performance SIBs. The rate performance of the GDOT electrode was evaluated at different current densities ranging from 0.1 to 30 A g^−1^. In Figure 6b, the GDOT electrode delivered high reversible specific capacities of 288, 261, 231, 208, 179, and 139 mAh g^−1^ at 0.2, 0.5, 1, 2, 5, and 10 A g^−1^, respectively. It should be noted that remarkable specific capacities of 95.0 and 62.3 mA h g^−1^ were sustained even at high current densities of 20 and 30 A g^−1^, respectively. In addition, a specific capacity of 192 mAh g^−1^ can still be returned when the current density is set back to 0.1 A g^−1^. As a comparison, bare GO electrode delivered reversible capacities of 104, 73, 56, 38 mAh g^−1^, 25 mAh g^−1^ at 0.2, 0.5, 1, 2, 5 A g^−1^, respectively and negligible reversible capacities (e.g., 10 mAh g^−1^ at 30 A g^−1^) at high current densities, which are much lower than those of the GDOT electrode. The cycling stability of bare GO, PEDOT, and GDOT hybrid for SIBs was also evaluated at 100 mA g^−1^ within the potential window of 0.005–3.0 V. In Figure 6c, a high specific capacity of 234 mAh g^−1^ can be maintained for GDOT after 400 cycles at 100 mA g^−1^, which is almost 2.4 times that of the GO electrode (97 mAh g^−1^). The pure PEDOT electrode shows fast capacity fading from 385 to 62 mAh g^−1^ during 200 cycles (Figure 7), which might originate from its large volume change. Based on the discussion, one can see that significantly enhanced rate capability and cycling performance are obtained for GDOT, which is due to the following several reasons: First, the hydrophilic GO can serve as an efficient reservoir for the electrolyte, and thus facilitates the migration of Na^+^. Furthermore, the uniform distribution of conductive fibers on GO surface significantly enhances the charge transfer by providing electron highway. In addition, the ultrathin GO as matrix can boost the accommodation ability towards large volume change.

To have a good understanding of the improved sodium storage properties, the EIS measurements were carried out after the third fully charged state. In Figure 6d, the semicircle in the high-middle frequency range corresponds to the charge transfer resistance (R_ct_) between the electrolyte and the electrodes. The low frequency line represents the Warburg resistance (Wo) related to the Li ion diffusion in electrode materials. From the fitted equivalent circuit in the inset of Figure 6d, the R_ct_ of the GDOT electrode (232 Ω) is much lower than that of the pure GO electrode (395 Ω), implying that the GDOT electrode possesses lower resistance. In addition, the CV measurements were also carried out on half cells at different scan rates from 0.1 to 1 mV s^−1^. In Figure 6e, the intensity of CV profiles for the GDOT electrode increases with the scan rate and shows no obvious distortion, indicating its quick CV response and fast reaction kinetics. According to the classical Randles–Sevchik equation (Equation (1)):

i_p_ = (2.69 × 10^5^)*n*^3/2^*SD*^1/2^*Cν*^1/2^ (1)

where *i_p_*, *n*, *S*, *D*, *C*, and *ν* represents the peak current (A), charge-transfer number, electrode area, diffusion coefficient of Na^+^ (cm^2^ s^−1^), concentration of sodium ions, and potential scan rate (V s^−1^), respectively. In addition, the above formula can be simplified to as:

i_p_ = *AD*^1/2^*Cν*^1/2^ (2)

where A is considered a constant for the three cells and *AD*^1/2^ can be supposed to be the apparent diffusion constant (ADC) of Na^+^. Through fitting the cathodic peak current and scan rate (Figure 6f), it can be found that GDOT electrode displays much higher apparent diffusion coefficient of Na^+^ with a slope of 0.38 compared to that of the pure GO electrode (0.1). The accelerated Na^+^ diffusion behavior of the GDOT electrode can be ascribed to the ultrathin Na^+^ transport pathway as well as electron highway originating from the well-designed conductive PEDOT fibers.

## 4. Conclusions

In summary, functional surface engineering of GO nanosheets towards enhanced sodium storage was realized by in situ polymerization of EDOT monomers under ambient conditions. Uniform PEDOT fibers can be deposited on GO within a short reaction time (40 min). As compared with the bare GO, GDOT exhibits high reversible capacity (338 mAh g^−1^), good cycling stability (234 mAh g^−1^ after 400 cycles) and rate capabilities (e.g., 62 mAh g^−1^ at 30 A g^−1^). The present results sheds light on developing high-efficiency carbon electrode materials for rechargeable batteries and also holds great potential for other energy storage devices.

## Supplementary Materials

The following are available online at http://www.mdpi.com/1996-1944/11/10/2032/s1, Figure S1: Cycling performance of the pure SWCNT electrode for SIBs at a current density of 100 mA g^−1^.
