# Supplementary material for: Poly (3,4-Ethylenedioxythiophene) (PEDOT) Nanofibers Decorated Graphene Oxide (GO) as High-Capacity, Long Cycle Anodes for Sodium Ion Batteries
Source: Materials (Basel). 2018 Oct 19;11(10):2032. doi: 10.3390/ma11102032 (PMC6213420; doi:10.3390/ma11102032)
Supplement: Supplementary file 1 [file materials-11-02032-s001.pdf]

Supporting Information

Poly (3,4-Ethylenedioxythiophene) (PEDOT) Nanofibers Decorated Graphene Oxide (GO) as High-Capacity, Long Cycle Anodes for Sodium Ion Batteries

Zejun Pu [1], Penglun Zheng [2,*] and Yu Zhang [3,*]

[1] College of Materials Science and Engineering, Sichuan University of Science & Engineering, Zigong 643000, China; puzj@susue.edu.cn
[2] High Temperature Resistant Polymer and Composites Key Laboratory of Sichuan Province, University of Electronic Science and Technology of China, Chengdu 610054, China
[3] School of Materials Science and Engineering, Nanyang Technological University, 50 Nanyang Avenue, Singapore 639798, Singapore
* Correspondence: 18482179228@163.com (P.A.); yzhang071@e.ntu.edu.sg (Y.Z.)

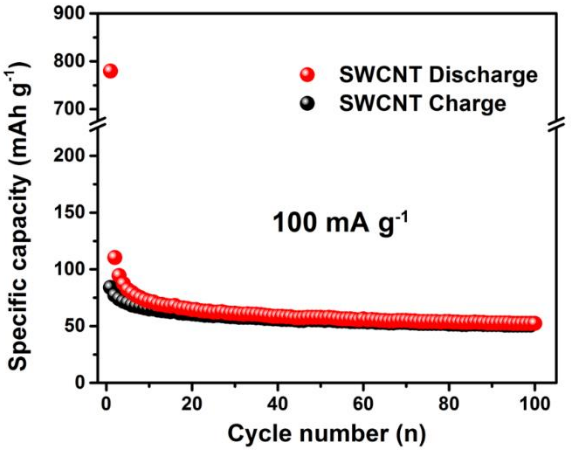

Figure S1. Cycling performance of the pure SWCNT electrode for SIBs at a current density of 100 mA g^{-1}.
